# A Crop Modelling Strategy to Improve Cacao Quality and Productivity

**DOI:** 10.3390/plants11020157

**Published:** 2022-01-07

**Authors:** Angela Patricia Romero Vergel, Anyela Valentina Camargo Rodriguez, Oscar Dario Ramirez, Paula Andrea Arenas Velilla, Adriana Maria Gallego

**Affiliations:** 1 The John Bingham Laboratory, The National Institute of Agricultural Botany NIAB, 93 Lawrence Weaver Road, Cambridge CB3 0LE, UK; 2 Federación Nacional de Cafeteros FEDECACAO, Calle 31 No. 17-27, Bogota 999076, Colombia; oscar.ramirez@fedecacao.com.co (O.D.R.); paula.arenas@fedecacao.com.co (P.A.A.V.); 3 Grupo BIOS, Centro de Bioinformática y Biología Computacional de Colombia—BIOS, Manizales 170001, Colombia; adriana.gallego.02@gmail.com

**Keywords:** thermal time, flowering date (FD), harvest prediction, biomass, light interception

## Abstract

Cacao production systems in Colombia are of high importance due to their direct impact in the social and economic development of smallholder farmers. Although Colombian cacao has the potential to be in the high value markets for fine flavour, the lack of expert support as well as the use of traditional, and often times sub-optimal technologies makes cacao production negligible. Traditionally, cacao harvest takes place at exactly the same time regardless of the geographic and climatic region where it is grown, the problem with this strategy is that cacao beans are often unripe or over matured and a combination of both will negatively affect the quality of the final cacao product. Since cacao fruit development can be considered as the result of a number of physiological and morphological processes that can be described by mathematical relationships even under uncontrolled environments. Environmental parameters that have more association with pod maturation speed should be taken into account to decide the appropriate time to harvest. In this context, crop models are useful tools to simulate and predict crop development over time and under multiple environmental conditions. Since harvesting at the right time can yield high quality cacao, we parameterised a crop model to predict the best time for harvest cacao fruits in Colombia. The cacao model uses weather variables such as temperature and solar radiation to simulate the growth rate of cocoa fruits from flowering to maturity. The model uses thermal time as an indicator of optimal maturity. This model can be used as a practical tool that supports cacao farmers in the production of high quality cacao which is usually paid at a higher price. When comparing simulated and observed data, our results showed an RRMSE of 7.2% for the yield prediction, while the simulated harvest date varied between +/−2 to 20 days depending on the temperature variations of the year between regions. This crop model contributed to understanding and predicting the phenology of cacao fruits for two key cultivars ICS95 y CCN51.

## 1. Introduction

Cacao (*Theobroma cacao* L.) is an important worldwide perennial tropical crop endemic to the South American rainforests [1,2,3,4]. Cacao plants are members of the Malvaceae (formerly Sterculiaceae) botanical family of which cotton *Gossypium hirstium* [5] is also a member. Cacao is grown for its fruits, known as cacao pods [6,7], which are mainly consumed as beverages. Only 5% of the world cacao yield is destined for fine-cacao production as most cacao farmers use outdated traditional crop management [3] methods that usually result in low crop productivity. In Colombia, cacao is one of the crops promoted by the Colombian government in the social and agricultural development programs aimed at favouring peace in post-conflict regions [4,8]. Cacao in Colombia is grown by approximately 52,000 rural families [9] of which 98% are small and medium-sized producers [10,11]. Colombia registered an increase of 3750 tons in cacao production in 2020 compared to the previous year [12].

Although Colombian cacao has the potential to be in the high value markets for fine flavour [11], the lack of expert support as well as the use of traditional, and often times sub-optimal, technologies makes cacao production negligibly. The farmer practice of empirically harvesting from five to six months, or 180 Days After Flowering (DAF) date, produces a mix of quality of cacao beans that are then fermented. The problem with harvesting at the wrong time is that cacao seeds are often either unripe or are beyond maturity and are already germinating inside the pod, both scenarios dramatically affect cacao quality. This harvesting practice also produces heterogeneous characteristics between each fermentation batch which potentially diminish the quality of the final cacao product [13]. In order to produce higher quality cacao beans, it is important to identify the best time to harvest, this prediction can be achieved by using simulation models that consider physiological responses affected by soil types, cultivar specifications and climatic factors (e.g., solar radiation and wind) are a useful tool to predict the right moment to harvest cacao pods.

In addition to quality, cacao productivity is already threatened by climate change [1,14] as rain and dry seasons are shifting and extending by several weeks; these changes in climate are ideal for diseases and pests to spread and thrive. Current adaptive responses to mitigate negative climate effects could be the use of tolerant varieties [14,15,16]. Statistical and mechanistic crop models which use weather data and other agronomic variables to predict growth can provide a tool to study the impact of climate change over cacao productivity. Crop models represent a quantitative assumption of plant growth depending on sunlight interception efficiency values and climate data supported by a large amount of empirical and ground data [17]. Physiological crop models have shown to be a very useful tool to provide agronomic advice to improve cropping systems of annual crops. However, most crop model studies focus on perennial crop production [1,18,19,20] and few on annual crops. The lack of models on annual crops can be due the difficulty to gathering field data, the relatively high research costs, and the difficulties of accumulated errors in long-term simulations [1]. Cacao yield prediction has been achieved by using machine learning [12] whose historic yield data are used to make future predictions or by using the “SUCROS-cacao” [1], a mechanistic model which simulates physiological cacao performance based on the estimation of light interception, photosynthesis, maintenance respiration, evapotranspiration, biomass production and yield. SUCROS-cacao is heavily parameterised with physiologic and morphologic data [1] which are usually region/variety sensitive and requires expert knowledge. To make harvest prediction simpler for farmers and developers, we adapted the simple generic crop model (SIMPLE) which uses few parameters, such as weather and cultivar specification [18] to simulate crop development and predict optimal pod harvest date and yield.

This paper reports the cacao model which is based on the physiological parametrisation of the SIMPLE crop model [18] to predict best harvest time and the potencial yield. Our aim was to calibrate a crop model that simulates crop development, growth and yield, and predicts the maturation day when the fruit is likely to be ready for harvest. Results can be a practical tool that supports cacao farmers in the production of high quality cacao, thus making Colombian cacao more competitive in the Fine-cacao market. This cacao model was developed as a product of KOCOLATL project, an international consortium between Colombian partners (research, farmers and organizations such as Fedecacao and BIOS) and UK partners (NIAB).

### Floral Phenology of Cacao

**Figure 1:**
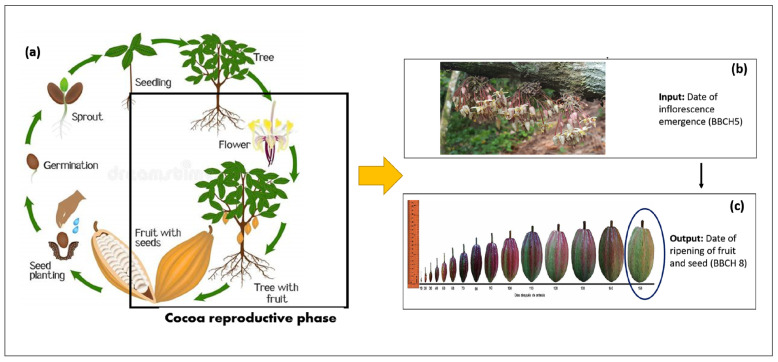
Phenology of cacao in Colombia for crop modelling. (**a**) flower phenology; (**b**) flowers at flowering date; (**c**) cacao fruit development) Source: Taken from Dreamstime.com phys.org (accessed on 21 November 2021) [21,26].

The phenological stages of a cacao tree are divided into two main phases, vegetative and reproductive. The reproductive phase (Figure 1a) is represented by the floral phenology which goes from the date of inflorescence emergence (BBCH scale 5) (Figure 1b) to the date of ripening of fruit and seed (BBCH 8) (Figure 1c) [6]. The reproductive phase is cyclically across two annual cycles which go through the following phases: inflorescence emergence, flowering, pollination, fruit development and harvest. Therefore, to model the cacao crop cycle as perennial plant, we regarded the start point of the cacao crop cycle as the inflorescence emergence date, as opposed to the sowing/emergence date usually used to model annual crops (Figure 1b). Therefore, the growth period of the fruit could vary from 110 to 150 daa (days after anthesis) [21] when cacao fruits reach the physiological maturity, but it can be harvested at 170 days daa [6] for quality purposes.

Since cacao is a cauliflorous plant, flowers grow on trunk and branches. Cacao trees usually produce up to 10,000 flowers per tree each year, 50% of which do not develop into ripe fruits (Personal communication with Fedecacao). Flower development takes approximately 30 days across 12 micro-stages, from meristem growth (stages 1 to 6) to the fully developed flower (Stages 7 to 12) [21,22] when it is ready to be pollinated. The opening of flowers or anthesis occurs over a 12-hour period during the night, and it is synchronised between the groups of mature flowers [6]. However, the life of a flower can last approximately one day after the opening, when it falls form the trunk if it is unfertilised [6,23]. Subsequentially, after anthesis, fruit growth for approximately 150 days until the maturation, mucilage. Therefore, the complete maturation process of the fruit, from the pollination to fully mature fruit, takes 160–210 days [24]. The accumulation of lipids, storage proteins and anthocyanin start about 85 days after pollination when fruits have an active metabolism and seed moisture content decreases up to 30% [6,25]. During this phase, the quality of cacao seeds is defined.

## 2. Materials and Methods

### 2.1. Test Site and Yield Production

**Figure 2:**
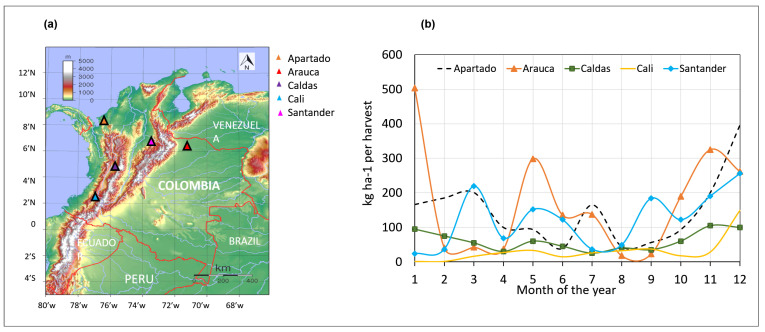
Cacao production of five farms in different regions. (**a**) map showing the regions where farms are located; (**b**) production per month (2019–2020) of five farms.

To calibrate and evaluate the cacao model, 23 field data samples per farm were collected, by Fedecacao, from five farms located in the geographic regions of Saravena (Arauca), Rionegro (Santander), Cali (Valle del Cauca), Apartado (Antioquia) and Manizalez (Caldas) (Figure 2a). Each field sample reported an observed flowering date and corresponding yield, for a given tree and month of the year. Field samples reported sights flowering dates from 12 July 2019 to 23 June 2020, each of these dates had their corresponding date of harvest after at exactly 180 DAF. Also, the age of trees, plant density (trees ha${}^{-1}$), yield (dry beans kg ha${}^{-1}$) and number of fruits harvested per hectare were reported. Thus, 23 flowering dates for five farms gave us 115 samples in total.

### 2.2. Weather Conditions

**Figure 3:**
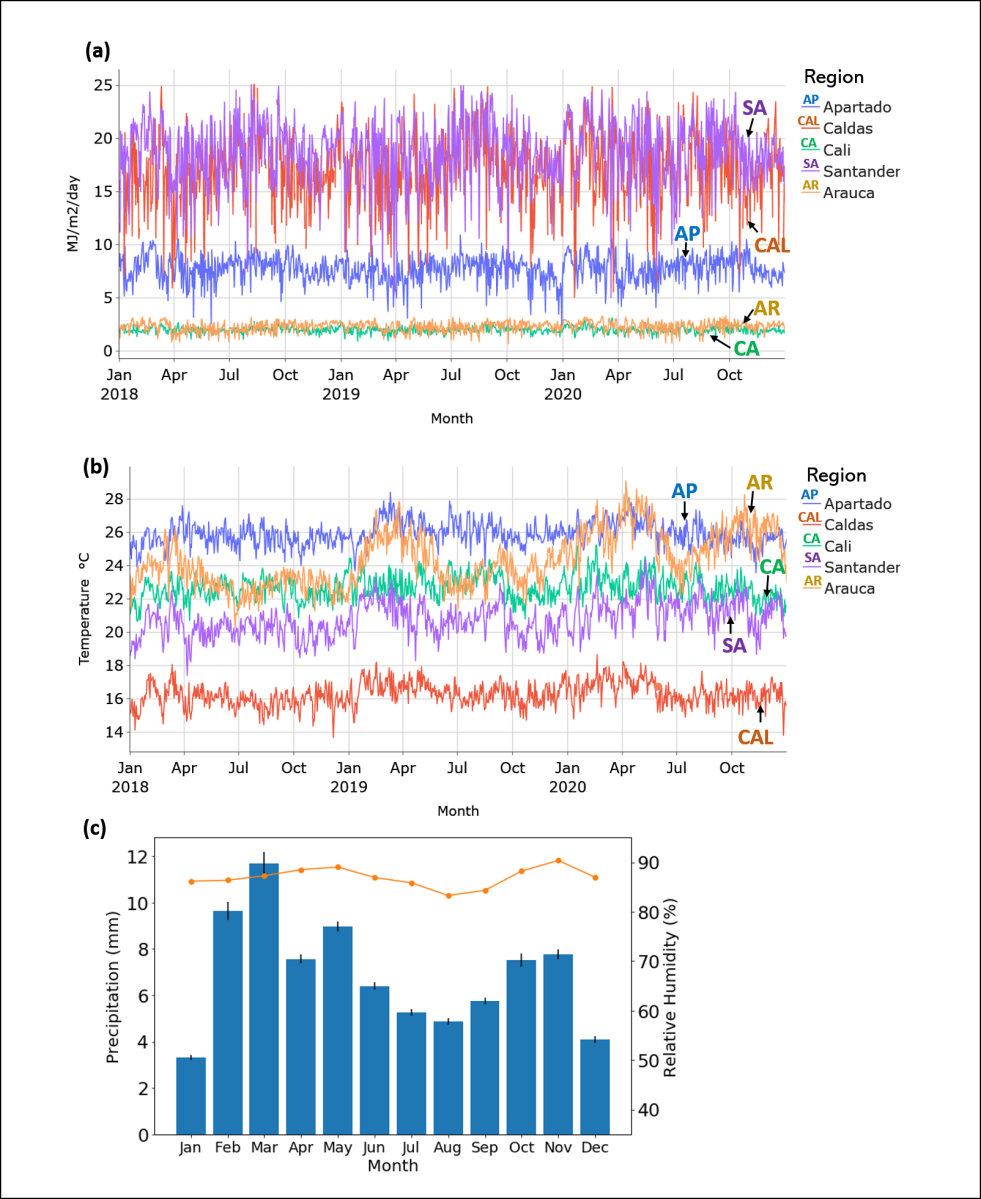
Colombian weather conditions. (**a**) available photosynthetic solar radiation (PAR); (**b**) daily average temperature; (**c**) monthly average of precipitation (bars) and relative humidity (dotted line) from 2018 to 2021.

We analysed and compared weather patterns that linked five geographical regions (Figure 2a) across 2018 to 2020. Looking at the data, the regions of Santander and Caldas had the biggest variability and the maximum of solar radiation values over 20 MJ m${}^{2}$day${}^{-1}$. In contrast, the regions of Cali and Arauca presented the lowest values of PAR below 5 MJ m${}^{2}$day${}^{-1}$. (Figure 3a). Even though Cali and Santander had contrasting PAR conditions, these regions presented have similar temperatures during 2020 (Figure 3b). The temperature ranges from 16 to 28 °C, and it is relatively constant for each region. However, Arauca presented the biggest variability with hotter months during the first half of the year 2019 and 2020. Apartado was found as the hottest region studied with a relative constant temperature of 26 °C and Caldas as the coldest site with 16 °C. Precipitation in Colombia is presented in two seasons per year from February to April and from October to November, while the relative humidity remains constant over 80% (Figure 3c). In general, the coldest regions tested (Caldas and Santander) had the maximum values of solar radiation available for photosynthesis.

### 2.3. Inputs and Data Acquisition

In order to model the phenology of the cacao fruit, linked to collected field data, the cacao model required specific input variables such as Flowering Date (FD), Daily Solar Radiation (SRAD), Daily Maximum and Minimum Temperature (TMAX, TMIN) and Daily Precipitation, specific to a particular agroclimatic region. FD was extracted from the field data, and corresponding weather data linked to a geographical region was sourced from the POWER Data Access Viewer [27], from 1 January 2018 to 31 December 2020 (Figure 2b). Weather data were processed using R (R 1.4 version) [28].

Since cacao yield depends on the successful development of flowers to form ripe pods, the current view from Colombian farmers (personal communication with Fedecacao) is that the highest flowering season occurs in September and January, which suggests that the harvest occurs in March and July. These communications were given through weekly online meetings of KOCOLATL project. However, the field data collected from Fedecacao suggested that flowering and pod production were not constant for all the locations. For example, pod harvest in the Caldas farm increased in May, and from October to December, and decreased from January to March. The Arauca and Apartado farms reported the highest yield in the months of January, July, November and December. The Santander farm had picks of production in March, May and September (Figure 2b).

### 2.4. Thermal Time for Pod Harvest Date Identification

The cumulative sum of daily temperature from a reference day 0 is defined as *Thermal time* and its units of measurement are in days degrees (days °C). That starter point of 0 days °C generally is the planting date [29], but, as indicated earlier, our cacao model used FD as the starting point. Thermal time for the development of a cultivated plant may consider the base temperature (T${}_{b}$), which is the minimum temperature required by cacao plants to grow. T${}_{b}$ can vary between cultivars [30,31]. For cacao, the vegetative growth T${}_{b}$ has been reported between 18.6 and 20.8 °C [31]. Nevertheless, the pod growth has a lower T${}_{b}$, which ranges between 9 and 12.9 °C [15,31]. We calculated the cacao thermal time with a pod growth T${}_{b}$ of 10 °C because it is the absolute minimum temperature for cacao growing in South America reported by [32] in [15].

The thermal time required for the cacao crop model was characterised for each location starting from FD (0 days °C) to harvest date (180 after flowering = 6 months), as farmers used to harvest by calendar days. Thus, the cacao model predicts the maturation day to harvest pods. It can vary depending on temperature variations. Thermal time was calculated using Equation (1), where tt is the cumulative sum of the daily temperature (T${}_{i}$) and T${}_{b}$ for cacao is 10 °C.

(1) $$Thermal\;time\;\left(tt\right)=\left\{\begin{array}{c} \sum_{i=1}^{n}T_{i}-T_{b}\\ \;\\ 0,\;Flowering\;date \end{array}\right.$$

### 2.5. Model Calibration

Calibration of any crop model is conducted typically for a particular cultivar and agroclimatic region [33]. The cacao model was calibrated, for five particular regions and two cultivars, by sequentially modifying the physiological variables and then comparing the degree of similarity between observed and predicted data [18,19,34,35]. The original code in R of SIMPLE model from [18] was modified into the cacao model. The process of calibration stops when the distance between observed and simulated data does not improve any longer. The cacao model has seven input csv files where new cacao crop data are provided. Files can be edited to define the features of the new cultivars or experiments. Since cacao phenology has not been simulated with the SIMPLE model, cacao physiological information such as Leaf Area Index (LAI) for shade plants [1,36,37,38] and Harvest Index (*HI*) [39] were extracted from the current literature and verified with Fedecacao cacao experts. Other variables such as Radiation Use Efficiency (RUE) [40,41] were based on the perennial crops banana and cotton which have been calibrated previously in the SIMPLE model [18] using RUEs of 0.8 and 0.85 for banana and cotton, respectively. As cacao trees usually grow under shadow [15], we estimated, by trial and error lower RUE values (between 0.7 and 0.5 g MJ${}^{-1}$ m${}^{2}$) (Table 1).

### 2.6. Parameters

This cacao model has three parameters which vary by region (Table 1): The thermal time required for harvest after the FD (Tsum), the Radiation Use Efficiency (RUE) and yield observed in the field. Physiological parameters in Table 2 are common for all the regions studied. These parameters were calibrated for cultivars ICS95 and CCN51 considering a range of time of 200 DAF to harvest day, even though farmers collect the pod at 180 DAF. Heat and water stress parameters were not considered as this study was not assessing biotic or abiotic stresses.

### 2.7. Evaluation of Model Performance

The cacao model performance was evaluated by comparing simulated values cacao yield with those reported by Fedecacao from cacao plantations, using the statistical index of Relative Root Mean Square Error (RRMSE) in Equation (2), where n is the total number of observations, Y${}_{i}$ corresponds to observed value from field and X${}_{i}$ is the predict value from the model:

(2) $$RRMSE=\sqrt{\frac{\frac{1}{n}\sum_{i=1}^{n}{\left(Y_{i}-X_{i}\right)}^{2}}{\sum_{i=1}^{n}X_{i}^{2}}}\times 100\%$$

## 3. Results

### 3.1. Weather Conditions over Flowering Time

**Figure 4:**
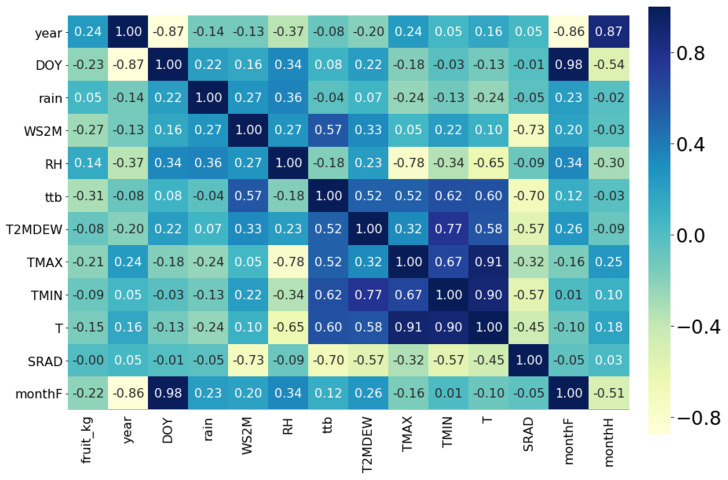
Pearson correlation average weather variables and FD for five locations in Colombia. Numbers in the squares are the correlation values.

Figure 4 shows the Pearson correlation to study the weather data of the flowering time over the months of flowering (monthF), month of harvest (monthH) and their final yield (fruit_kg). The results showed that thermal time T${}_{b}$ (ttb) is correlated (*r* = 0.52) with daily average temperature and maximum temperature (TMAX) and temperature minimum (TMIN) and Dew Frost Point at 2 m (T2MDEW) with a correlation coefficient of *p* = 0.60. The wind (WS2M) is correlated with ttb with *p* = 0.57. However, less clear correlations were found for monthF with T2MDEW, relative humidity (RH), WS2M and rain. However, farmers stated that the number of flowers pollinated decrease by months where wind and rain are high (personal communication with Fedecacao), and our analysis (Figure 4) could not show a correlation between flower shedding and high wind or rain because information on number of flowers at FD was not part of the field data collected.

### 3.2. Thermal Time

**Figure 5:**
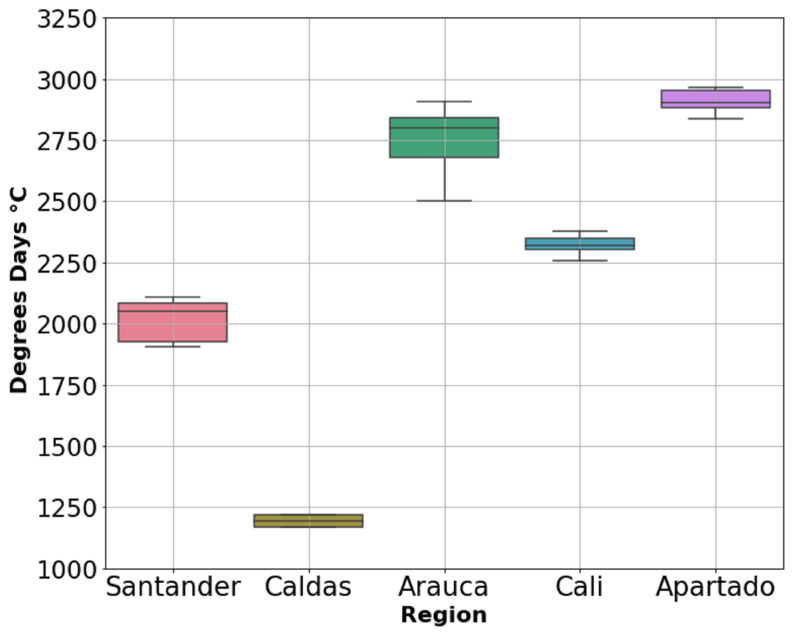
Cacao yield and thermal time characterisation at 180 DAF.

Thermal time characterisation was made considering 180 DAF for each location. The box-plot in Figure 5 shows the data distribution where boxes indicate the range of the central 50% of the total data per region, the central line in the box is marking the median value and lines draw out from each box mean the range of the remaining data. Therefore, this boxplot shows differences between locations as was expected following the tendencies of the temperature of Figure 3b, where Aparatado, Arauca and Cali had greater temperatures than Santander and Caldas.

Therefore, Apartado and Arauca had the highest temperatures and consequently the highest thermal time values with 2909 and 2764 days°C, respectively. Caldas had the lowest values with 1173 days°C. Meanwhile, Cali and Santander presented similar thermal time around 2000 °C (Table 1). The accumulated temperature during the pod development (Figure 5) depends on the region where cacao is cultivated and the variety planted. Thermal time values are also proportional to the yield reported on field (Table 1).

### 3.3. Model Validation

Biomass production of the aerial part of the cacao tree was simulated which includes every organ of the plant that is over the soil surface. It is important to calculate how much biomass from the crop aerial is partitioned to the cacao pods according to the harvest index (*HI*) (Table 2) as this quantity will correspond to the weight of cacao seeds.

**Figure 6:**
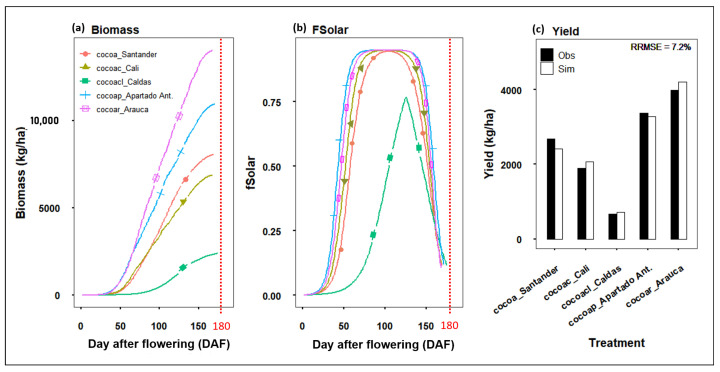
Model predictions. (**a**) biomass aerial part; (**b**) interception of solar radiation; (**c**) yield crop cycle close to 180 DAF (vertical red line) based on Figure 5.

Figure 6a shows the daily biomass growth rate for five agroclimatic regions. Biomass simulation is affected by the solar radiation, RUE [40,41], daily temperature, atmospheric CO${}_{2}$ concentration (ppm) and the fraction of solar radiation intercepted by a tree of cacao during the fruit development (fSolar) (Figure 6b). We did not compare predicted and observed Biomass as there were not field data corresponding to absorption of solar radiation by the plants or of biomass production. Figure 6c also shows that the daily biomass growth rate increased proportionally with the yield production, suggesting that it was possible to calculate the final yield as the product of accumulated biomass at the harvest day and *HI* = 0.3 (Equation (3)):

(3) cacaoyield=Accumulatedbiomass×HI

The fraction of solar radiation intercepted by cacao trees (fSolar) was also simulated for the fruit development cycle. The relation of fsolar is strongly related with RUE [40,41], LAI and hence the senescence of the canopy leaves [15,18,37,42,43,44]. Results in the Figure 6b showed that all regions had a maximum fSolar of 0.94, except from Caldas whose peak fSolar was at 0.76. fSolar-max was more quickly reached in the regions of Apartado and Arauca. These fSolar-max values of solar radiation intercepted for photosynthesis lasted differently depending on the region and their solar radiation (Figure 3a): Apartado 66 days from 69 to 135 DAF, Arauca 61 days from 72 to 133 DAF, Cali 38 days from 83 to 121 DAF, Santander 24 days from 92 to 116 DAF and Caldas 2 days at 125 DAF. fSolar declined and hence the interception of solar radiation until the pod harvest day.

The RRSME has been used before to evaluate other crop model simulations [18,19]. Our results of cacao yield simulation (kg ha${}^{-1}$ per year) and validation (Table 1) achieved a final RRMSE of a 7.2% fit between simulated and observed data (Figure 6c). The low RRMSE indicates the simplicity of the Cacao model to simulated cacao yield. However, the original SIMPLE model can be used to assess heat and water stress, and our cacao model calibration did not account for biotic (pest and diseases) and abiotic (heat or water stress) factors that could have significant effects on the cacao production.

Individual errors per region are presented in Table 3, where the best fit of the calibration model for yield prediction was for crops in Apartado, and the highest error was calculated for Caldas crops. The model responded to the variations of temperature and solar radiation. Therefore, the highest yield values simulated were obtained for Arauca over 4000 kg ha${}^{-1}$, followed by Apartado Santander with yields over 2000 kg ha${}^{-1}$. The lowest yield was simulated for the Caldas region with less of 1000 kg ha${}^{-1}$. Final yield in the model was calculated as the product of biomass of aerial part and harvest index (*HI*) [18,45], where the *HI* is similar to the CropSyst [46] and AquaCrop [47].

### 3.4. Predicting Optimal Pod Harvest Day

**Figure 7:**
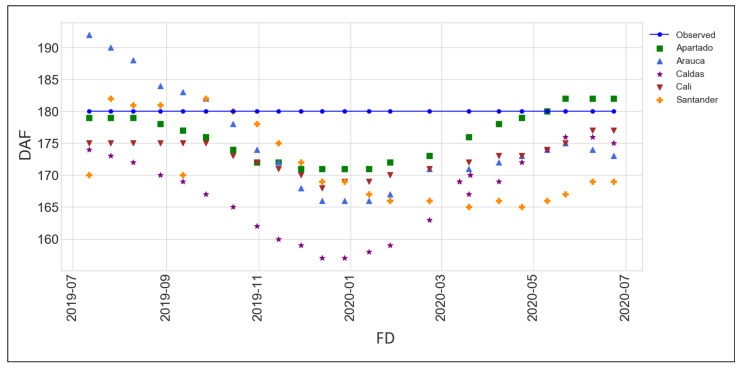
Cacao harvest day prediction from FD for Apartado, Arauca, Caldas, Cali and Santander. DAF = Days After Flowering. FD = Flowering Date.

Farmers’ practice of empirically harvesting is about counting 180 DAF (6 months) by calendar without taking into account weather changes or physiological features of cacao cultivars. Since our cacao model was fitted to predict optimal pods’ harvest dates, we compared model predicted dates against the usual 180 days that growers and experts count after the FD. Figure 7 shows the observed day of harvest (180 DAF) independently of the region. All the regions except Santander presented the earliest predicted harvest when the FD was between December and February. The fruit can be ready to harvest before or after 180 DAF depending on the environmental condition per region. The results in Figure 7 demonstrated that the traditional way to harvest, which is always at 180 DAF, is not taking into account physiological and environmental conditions that can be affecting the pod maturation. Thus, when the fruit development was simulated, the maturity day in Cali and Caldas were from 3 to 12 and 4 to 23 days before 180 DAF. Apartado presented the most similar predicted dates of harvest to 180 DAF with 170 to 182 DAF. The pod may be harvested in Arauca from 165 to 193 DAF, Santander from 165 to 183 DAF, and ten days less than in Arauca for the same months of flowering of July and August of 2019. Only Arauca presented longer crop cycles when the FD was between July and September of 2019. This means that Arauca had bigger variation of temperature between months. To summarize, depending on which month of the year cacao trees are flowering, the number of days to reach the harvest of ripe pods vary more or less 180 days. For example, if cacao trees in Caldas are flowering in January, pods will be ripe to harvest in approxmately 158 days (Table 4).

## 4. Discussion

This study reports the cacao model which was calibrated for five agroclimatic regions in Colombia. The model simulates crop development, growth and yield, and predicts the maturation day when the fruit is likely to be ready for harvest. The purpose of this model was to help farmers achieve higher quality cacao beans by providing them with a tool that not only predicts optimal harvest date but also estimated a potential yield and biomass. The low RRMSE for yield prediction indicates the simplicity of the Cacao model to simulated cacao yield. Although the original SIMPLE model can be used to asses heat and water stress, our cacao model calibration did not account for biotic (pest and diseases) and abiotic (heat or water stress) factors that could have significant effects on the cacao production. Santander and Caldas have the highest solar radiation but the lowest temperature. In contrast, Arauca and Apartado have the highest temperature but the lowest solar radiation. Weather analysis could not show a correlation between flower shedding and high wind or rain because information on flowers was not part of the field data collected. The growth cycle was simulated in terms of thermal time which was defined for the five regions tested, given the dependency of the crop model to predict the harvest day according to the weather variations while fruit are growing. The thermal time calculation was defined based on 180 DAF because farmers cut the pods by calendar days. Our results showed how the harvest day can vary depending on the accumulate temperature during each specific crop cycle simulated (Figure 7).

### 4.1. Weather Effects over Flower Stability and Pollination

Our analysis of weather variables (Figure 4) could not show a correlation between flower shedding and high wind or rain because information on flowers was not part of the field data collected. However, farmers stated that the number of flowers pollinated decrease by months where wind and rain are high. High winds can affect the availability of tiny flies pollinators from Diptera order and from the families of of the biting midges *Ceratopogonidae*, genus *Forcipomyia* [48,49,50] to reach the cacao flowers. However, the stability of cacao flowers is influenced by seasonal weather conditions (abiotic) and pollination (biotic) [51]. Therefore, pollinator population should be coincident with the phenology of the flowering cacao trees [52,53]. Flower opening is very well synchronised between the cohorts of mature flowers opening each night [6]. The flowers open at almost exactly the same time and rate, irrespective of their position on the trunk. Thus, unfertilised flowers abscise from the trunk approximately one day after flower opening [6]. Hence, more than 90% of unpollinated flowers fall or abscised within 32 h after anthesis [54]. Abscission processing of flowers is mainly controlled by three hormones: auxin, ethylene, and abscisic acid (ABA) [54]. Ethylene generally promotes abscission because it may inhibit the transport of auxin from the leaf blade, which allows the action of ABA to promote the fall of flowers [55]. In general, environmental conditions can also stimulate a decrease in the auxin/ethylene relationship [54].

When analysing the data from Santanter region, we showed that the number of successful flowers pollinated to produce final yield could be affected by rain, TMAX and wind. Nevertheless, better field data tracing flowers development is essential to understand if it is a mechanical or physiological effect. Refs. [56,57] indicated that the numbers of cacao pollinators were reduced during the dry season but increased in the wet season. This could be due to midges needing a moist environment to develop [51], which is difficult during the dry season as cacao leaves create a dried ground mat [51,56]. Moreover, the lack of water during dry seasons may reduce the nutrient uptake, provoking the massive flower drops [58]. For future studies, wind could be included in the cacao model as an input to simulate these mechanical effects over the number of flowers. In general, field data regarding counting flowers pollinated by month should be better reported for the region studied here.

### 4.2. Thermal Time for Harvest Day Predictions

We define the thermal time required to harvest cacao pods for five Colombian regions as maturation of the fruit is related with temperature during the growth cycle [21]. Previous studies have calculated thermal time for different cacao cultivar in Brazil and Ghana [31]. They also confirm that the fruit maturation time decreases with an increase in temperature as was presented in other research [31,59,60]. The effects of temperature and solar radiation on fruit growth and development were previously studied by [31], showing that crops in higher temperatures induce greater fruit losses because of physiological maturation (cherelle wilt). When the fruit is mature, seeds are able to germinate [21]. However, when ripe fruits stay for a longer time on the tree without being harvested at the right moment, seeds can germinate inside the pod, damaging cacao production for high-quality taste and affecting the final yield.

Our results showed that the hotter regions such as Apartado and Arauca presented higher thermal time values (Figure 3 and Figure 5). Even though Arauca had very low values of SAR but very high temperatures, this may be caused by cloud cover. The opposite can be seen for Caldas and Santander. These extreme relations T/SAR can compensate the crop efficiency, for example in Santander (Figure 6). The thermal time calculation was defined based on 180 DAF because farmers cut the pods by calendar days. Previous studies stated that evaluating the level of knowledge of growers regarding cacao crop management showed that the harvest was in the group of activities that presented the lowest level of information by the farmers [9]. This is why these results present important temperature boundaries to predict fruit maturation day. Therefore, there may be other environmental factors that should be studied for further research.

### 4.3. Cacao Crop Model Simulations

Although cacao is a relevant crop and there is an extensive agronomic literature, there is only one physiological crop model specific for cacao so far, which is SUCROS-cacao developed by [1]. However, the code was not easy available for adaptations. In contrast, the SIMPLE model has an open code in R, and if we could adapt such a model, it would be very useful to compare yields and predict harvest date in different climates. As the harvest day was predicted from the FD (Figure 7, consequently, biomass production and fSolar presented a crop cycle shorter than 180 DAF (Figure 6a,b). These simulations are coincident with results presented by [21], where physiological maturity of coca pod varies from 140 to 162 DAF. Our results showed how the harvest day can vary depending on the accumulate temperature during each specific crop cycle simulated (Figure 7). If cacao fruits are harvested at the right moment, the quality of the seed inside the pod can improve avoiding the germination before collecting the pod from cacao trees. Therefore, the cacao model can predict with more detail the optimum day to harvest the pod, ensuring the quality of the cacao bean destined for fine-cacao products. Fruit ripening is a highly coordinated developmental process that coincides with seed maturation [61]. As cacao is a non climacteric fruit, the pod needs be connected to the mother plant until maturation. Therefore, an indirect way to infer the seed quality is checking the pod quality. Pod quality can be determined as the physico-chemical changes occurring at the peel level, (including coloration changes, and chemical dynamical changes in metabolites production such as sugars, phenolics, and fatty acids) which give information about the seed status. For instance, clear evidence of the above occurs when cacao ripe fruits stay for a longer time on the tree without being harvested on the right moment, causing seeds to germinate inside the pod and damaging the cacao production for high-quality taste and affecting the final yield. An ongoing project conducted by BIOS is showing us the expression patterns for metabolites associated with cacao ripening. In this case, we found sugars, phenolic, fatty acids and flavonoids, which is in accordance with the ripening stage change their expression. Furthermore, we are identifying biochemical markers of fruit ripening. We are also connecting these chemical changes with seed quality. It lets us understand how the ripening occurs in cacao and suggests the best moment to harvest.

Biomass simulations use the cacao model presented similar predicted values (10,000 Kg ha${}^{-1}$) for coca drops in Costa Rica using SUCROScacao [1]. Biomass simulations are a common evaluation in crop models such as Sirius [33], SUBSTOR-potato [62] and DSSAT, CropSyst, STICS and WOFOST [63]. The approach of this research was focused on the harvest date prediction, hence the leaves of trees during the crop cycle was evaluated indirectly in this study because the fraction of intercepted photosynthesis active radiation (fSolar) decreased (Figure 6b) when the senescence of the canopy occurred [1]. The relation of fsolar and LAI [15,37,38,42,43,44] and RUE [40,41] has been widely reviewed in literature. The production of photo-assimilates, dry matter and yield can be affected by LAI and RUE reduction [40,41,43,64]. Therefore, LAI values are utilized to predict primary photosynthetic production and crop growth [1,37,38,43]. In cacao canopy, senescence referred to a group of leaves responsible at the moment of the fruit formation for producing carbohydrates. These leaves eventually drop becoming litter over the soil. Leaf life cycle has been simulated using crop models [1,65], which can be the reference to improve our cacao crop model in future studies.

Yield prediction presented a RRMSE values 7.2%, which were significantly lower than those presented for other crops using the SIMPLE model which reported an RRMSE of 24.4% [18], resulting in a reliable approach for cacao yield prediction. The low RRMSE for yield prediction indicates the simplicity of the cacao model to simulate cacao yield. Although the original SIMPLE model can be used to asses heat and water stress, our cacao model calibration did not account for biotic (pest and diseases) and abiotic (heat or water stress) factors that could have significant effects on the cacao production. In general, these results may help to improve the quality of cacao seed considering that the moment to harvest can be variable depending on weather changes.

### 4.4. App Development

The cacao model code was adapted to make the implementation of this cacao model easy, and app can be used in smartphones and desktops by farmers in Colombia. Therefore, the new version for cacao crops simulation will be used to predict yield, date of harvest and biomass production, inserting only the date of flowering and region. Grupo BIOS is in charge of app development, which is scheduled to be delivered to farmers in Caldas initially at the end of 2021.

## 5. Conclusions

Optimal harvesting time of cacao pods is one of the best strategies to obtain highly homogeneous cacao beans that are mature enough to produce high quality cacao. Predicting the right time to harvest cacao pods depends on multiple factors that can be used to model the physiology and phenology of the crop, over time and climatic environment. Our cacao model allowed for predicting the harvest date with better precision than only considering days by calendar, applying the thermal time characterisation, which ranges from 1200 to 3000 days${}^{\circ }$C, with a T${}_{b}$ of 10 ${}^{\circ }$C for the fruit development. Our model suggests that the crop cycle of cacao for harvest should be shorter than 180 days after flowering.

Although the results of this study were based on the analysis of five Colombian regions, the model can be easily calibrated for other geographic regions outside Colombia, in addition to other cacao varieties. What is really essential is to have good quality data that can be used to calibrate and test the models. In addition to using weather as a predictor of optimal harvest time, the model reported here can be extended to include the effect of diseases, nutritional deficiencies and abiotic stresses over pod production. Ideally, farmers use models as tools to plan ahead and be prepared for the disruption caused by changes in climate.

## Figures and Tables

**Table 1:** Cacao crop parameter values used per region.

Region	Tsum	RUE	Yield *
Apartado	2906	0.6	3378
Arauca	2764	0.7	3981
Santander	2016	0.6	2687
Cali	1912	0.5	1900
Caldas	1192	0.6	740

RUE Radiation use efficiency (above ground only and without respiration)g MJ^−1^ m^2^. * Yield observed kg ha^−1^ per year.

**Table 2:** Parameter values used to run the cacao model.

File	Variable Name	Value
	SoilName	Loamy sand4
	InitialFsolar	0.01
Treatment	Weather	KOKO (.WTH file name)
	CO${}_{2}$	400 ppm
	SowingDate	Flowering Date (FD)
	Crop cycle DAP	200 days
	LAI	1.8
Observation	FSolar	0.70
	Biomass	40 kg dry mass per plant
	Harvest index	0.3
Cultivar	150A	680 °C day
	150B	680 °C day
	Tbase	10 °C
	Topti	26 °C
Species	MaxT	35 °C
	ExtremeT	40 °C
	CO${}_{2}$RUE	0.09 °C
	S-water	0 ARID index

S-water is associated drought stress evaluations ranging from 0 (no water shortage) to 1 (extreme water shortage) [18].

**Table 3:** Summary of relative root mean square error RMSE for yield prediction using the cacao model.

Region	Apartado	Arauca	Santander	Cali	Caldas	Overal
RMMSE%	3	6.05	10.06	8.5	14.90	7.2

**Table 4:** Average of days to harvest according to the month of flowering.

Month	Santander	Arauca	Cali	Apartado	Caldas
January	166.5	166.5	169.5	171.5	158.5
February	166	171	171	173	163
March	165	171	172	176	167
April	165.5	172.5	173	178.5	170
May	166.5	174.5	174.5	181	175
June	169	173.5	177	182	175.5
July	176	191	175	179	173.5
August	181	186	175	178.5	171
September	176	182.5	175	176.5	168
October	179	176	172.5	173	163.5
November	173.5	170	170.5	171.5	159.5
December	169	166	168.5	171	157

Days to harvest cacao after flowering are approximate, as these are results from the cacao model simulations. Calibration was based on FEDECACAO reports from 2018 to 2020.

## Data Availability

Software will be provided upon user request. The data used in this study can be found as follows: kocolatl is available in the research data repository. https://github.com/anyelacamargo/kocolatl.git accessed on 15 December 2021, UK.
